# Supplementary material for: Scaled laboratory experiments explain the kink behaviour of the Crab Nebula jet
Source: Nat Commun. 2016 Oct 7;7:13081. doi: 10.1038/ncomms13081 (PMC5059765; doi:10.1038/ncomms13081)
Supplement: Supplementary Figures and Table. — Supplementary Figures 1-4 and Supplementary Table 1. [file ncomms13081-s1.pdf]

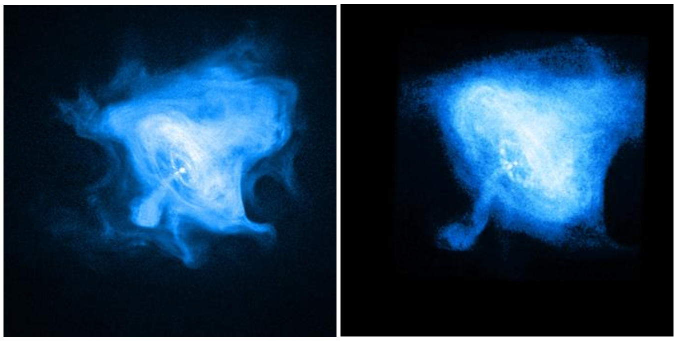

November 5, 2008 May 11, 2011

Supplementary Figure 1. X-ray images of the South-East jet in the Crab nebula. (http://chandra.harvard.edu/photo/2009/crab/). Taken by the Chandra X-ray Observatory, the considerable deflection of the jet propagation during this period of time indicates such a jet changes direction every few years.

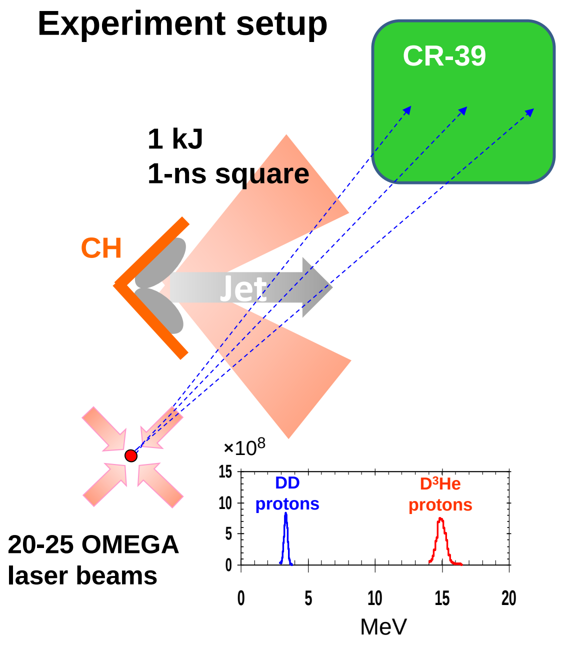

Supplementary Figure 2. Experiment setup. The proton backlighter (an imploded D^3He-filled thin-glass-shell capsule driven by 20 - 25 OMEGA laser beams) is 1 cm from the jet and has the illustrated monoenergetic proton lines from the reactions D + ^3He → α + p (14.7MeV) and D + D → T + p (3.0 MeV). The distance from jet to the imaging detector is 27 cm.

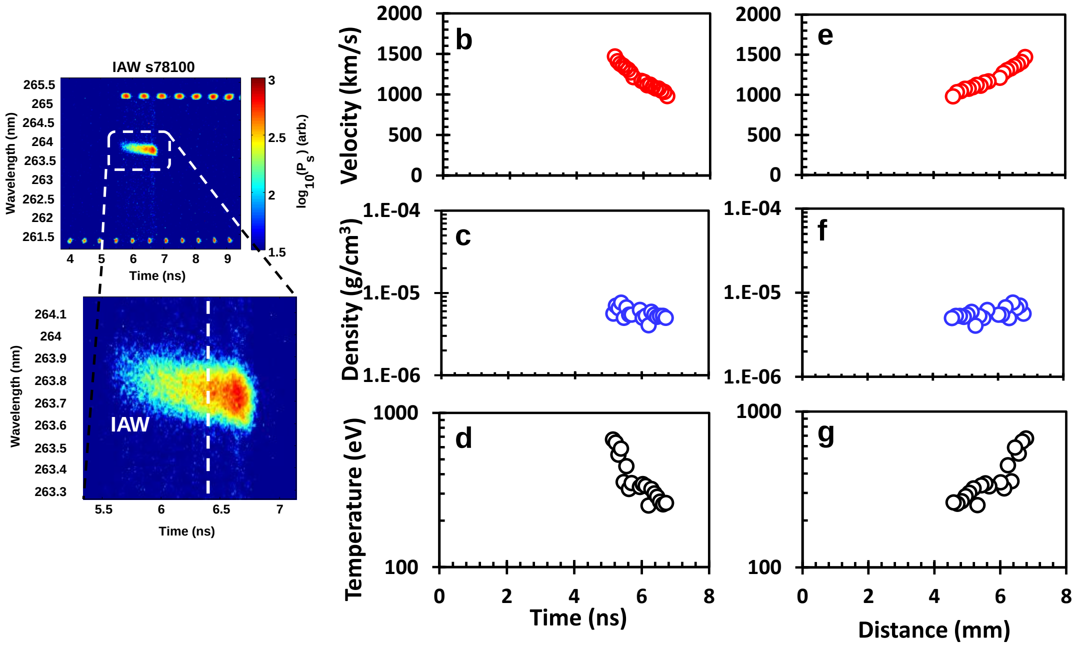

Supplementary Figure 3. 4ω Thomson scattering measurements. (**a**) Thomson-scattering spectra of ion-acoustic wave measured at position z = 7 mm. The white dashed line in the enlarged image shows a sample lineout. In these experiments, the plasma density was too low for direct T_e measurement, but the Thomson-scattering data show plasma ion-acoustic waves whose properties were fit with a multi-parameter model that determined T_e as shown (assuming $T_i \sim T_e$). The equality of T_i and T_e makes sense, because the jet was collisional with electron mean-free-paths much smaller than the jet radius [λ_{mfp} (~ 10 μm) << r_j (~ 500 μm)]. As indicated by the simulation, while T_i was higher than T_e close to the jet-launching region (z ~ 2 mm) because the heating from the collision of the two plasma plumes, the e-ion equilibrium time is shorter than the time preceding the measurements ($\tau_{e\text{-}ion}$ ~ 800 ps << t ~ 5 ns), leading to jet plasma thermalization and e-ion equilibrium. In the left column, a number of physical properties of the jet at position z=7 mm are plotted as a function of Thomson probing time (uncertainty $\Delta t = \pm$ 50 ps): (**b**), jet flow velocity (uncertainty $\Delta v = \pm$ 14 km s^{-1}); (**c**), jet plasma density ($\Delta \rho = \pm$ standard deviation); (**d**), jet plasma temperature ($T_e \sim T_i$, $\Delta T = \pm$ 50 eV). In the right column, jet velocity, (**e**), plasma density, (**f**) and plasma temperature, (**g**) inferred at probing time $t = t_0 + 5$ ns are plotted as a function of the jet position ($\Delta z < \pm$ 0.1 mm), respectively (the error bar are same as corresponding parameters in left column).

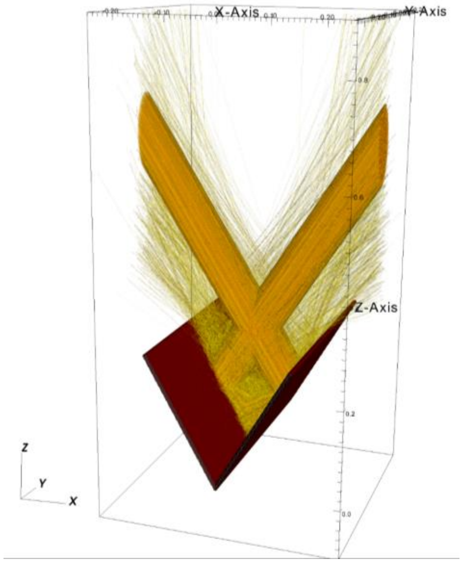

Supplementary Figure 4. Initial setup for the 3D FLASH simulation. Each individual foil was driven by two laser beams (0.351 μm in wavelength) at an angle ~ 28º to the foil normal, with a total energy ~ 1000 J in a 1-ns, square-top laser pulse with full spatial and temporal smoothing.

Supplementary Table 1. Summary of a typical proton backlighter (thin-glass shell capsule)

Capsule diameter	~ 420 μm
Capsule shell thickness	~ 2 μm
Fuel (filled gas)	~ 18 atm D^3He gas
Total laser energy	~ 10 kJ
Fuel ion temperature	~ 10 keV
D^3He proton energy	14.7 MeV
D^3He proton yield	~ 1×10^9
DD proton energy	3 MeV
DD proton yield	~ 1×10^9
Nuclear bang time	~ 400 ps
Nuclear burn duration	~ 100 ps
Nuclear Burn size (FWHM)	~ 40 μm
